# Snapshots of ligand entry, malleable binding and induced helical movement in P-glycoprotein

**DOI:** 10.1107/S1399004715000978

**Published:** 2015-02-26

**Authors:** Paul Szewczyk, Houchao Tao, Aaron P. McGrath, Mark Villaluz, Steven D. Rees, Sung Chang Lee, Rupak Doshi, Ina L. Urbatsch, Qinghai Zhang, Geoffrey Chang

**Affiliations:** a Division of Biological Sciences, University of California at San Diego, La Jolla, CA 92023, USA; b Department of Integrative Structural and Computational Biology, The Scripps Research Institute, La Jolla, CA 92037, USA; c Skaggs School of Pharmacy and Pharmaceutical Sciences, The Scripps Research Institute, La Jolla, CA 92037, USA; d Skaggs School of Pharmacy and Pharmaceutical Sciences, The University of California, La Jolla, CA 92023, USA; e Cell Biology and Biochemistry, Texas Tech University Health Sciences Center, Lubbock, TX 79430, USA; f Department of Pharmacology, School of Medicine, University of California at San Diego, La Jolla, CA 92093, USA

**Keywords:** P-glycoprotein

## Abstract

P-glycoprotein (P-gp) is a transporter of great clinical and pharmacological significance. Several structural studies of P-gp and its homologs have provided insights into its transport cycle, but questions remain regarding how P-gp recognizes diverse substrates and how substrate binding is coupled to ATP hydrolysis. Here, four new P-gp co-crystal structures with a series of rationally designed ligands are presented. It is observed that the binding of certain ligands, including an ATP-hydrolysis stimulator, produces a large conformational change in the fourth transmembrane helix, which is positioned to potentially transmit a signal to the nucleotide-binding domains. A new ligand-binding site on the surface of P-gp facing the inner leaflet of the membrane is also described, providing vital insights regarding the entry mechanism of hydrophobic drugs and lipids into P-gp. These results represent significant advances in the understanding of how P-gp and related transporters bind and export a plethora of metabolites, antibiotics and clinically approved and pipeline drugs.

## Abstract 2

Co-crystal structures of P-glycoprotein with a series of engineered ligands reveal multiple ligand-binding modes, a ligand-binding site on the outer surface of the transporter and a conformational change that may couple to ATP hydrolysis.

## 1. Introduction   

Membrane-embedded transporter proteins mediate the passage of metabolites and toxins across cellular membranes. The transporter P-glycoprotein (P-gp; ABCB1; EC 3.6.3.44) is expressed in the intestines, liver, kidney and blood–brain barrier, and affects the bioavailability, pharmacokinetics and efficacy of drugs (Cascorbi, 2006 ▶). P-gp also causes cellular multidrug resistance, hindering the treatment of several diseases, including cancers and HIV (Eckford & Sharom, 2009 ▶; Gottesman & Ling, 2006 ▶; Falasca & Linton, 2012 ▶). As such, the US Food and Drug Administration (FDA) now mandates the documentation of P-gp–drug interactions for the approval of any new drug (Giacomini *et al.*, 2010 ▶; US Food and Drug Administration, 2012 ▶). Thus, the inhibition or evasion of P-gp without compromising therapeutic efficacy is a major goal of the pharmaceutical industry.

P-gp belongs to a superfamily of ATP-binding cassette (ABC) transporters found throughout all kingdoms of life. The transporter consists of two pseudosymmetric halves encoded into a single polypeptide. Each half is formed by six transmembrane helices (TMs) and one cytosolic nucleotide-binding domain (NBD) along with interconnecting loops and short helices. The two structural halves enclose a central pocket/cavity which contains multiple discrete binding sites for ligands of P-gp, drugs and transport substrates. P-gp and related ABC transporters drive substrate translocation using ATP binding and hydrolysis at the cytosolic NBDs. Because P-gp can transport a wide array of structurally diverse compounds, a molecular framework for understanding how different ligands enter and bind is crucial to overcoming P-gp-mediated drug efflux.

The crystallography of protein–ligand complexes can be a challenge at low to moderate diffraction resolutions. For co-crystals comprising transporters in complex with ligands of relatively low binding affinity (in this case the transport substrates), the level of difficulty in resolving their positions increases. Our previous structures of P-gp bound to two enantiomeric cyclopeptides provided the first structural view detailing how P-gp recognizes and binds ligands (Aller *et al.*, 2009 ▶). In that study, we confirmed the presence of selenium-labelled ligands using anomalous diffraction methods. Unfortunately, the lower resolution of those structures (4.4 and 4.35 Å) limited the interpretation of ligand–P-gp interactions. Here, we report higher resolution (3.4–3.8 Å) structures of P-gp bound to a series of rationally engineered selenium-labelled cyclopeptide compounds that were designed to probe the ligand-binding pocket of P-gp. In doing so, we reveal how drug substrates (i) may enter the transporter, (ii) bind at multiple, shared yet discrete sites in the transmembrane domain (TMD) and (iii) might transmit this information to the NBDs to stimulate ATP hydrolysis. Together, these new structures provide snapshots of how structurally similar ligands, differing only in side-chain *R*-group size, can bind prior to drug efflux. These higher resolution P-gp structures also resolve, to an extent, the issue of TM registry (Jin *et al.*, 2012 ▶; Li *et al.*, 2014 ▶).

## 2. Materials and methods   

### 2.1. Synthesis of QZ-homotrimers   

*N*-Boc-(*S*)-amino-acid selenazole esters were prepared from the corresponding *N*-Boc-(*S*)-amino acids according to a previously published procedure (Tao *et al.*, 2011 ▶). The selenazole ester derived from *N*-Boc-(*S*)-Ala (500 mg, 1.44 mmol) was subjected to hydrolysis by a solution of NaOH in mixed solvents (5:1:2 THF:MeOH:H_2_O) followed by Boc deprotection by 50% trifluoroacetic acid in dichloromethane. The resulting amino acid (∼1.4 mmol) was dissolved in anhydrous acetonitrile (14 ml) and treated with diisopropylethylamine (DIEA; 1.0 ml, 6.1 mmol) and pentafluorophenol diphenylphosphinate (FDPP; 1.1 g, 2.9 mmol). The reaction was stirred at room temperature for 24 h before being concentrated under vacuum. The residue was dissolved in dichloromethane and washed successively with NaHCO_3_, 5% HCl aqueous solution and NaCl solution. Organic phases were concentrated and the residue was purified by silica-gel chromatography. The major product was collected and identified as the desired product QZ-Ala (92 mg, 32%).

The other cyclopeptides were obtained by following the same procedure. The purity of the individual cyclopeptides was >98% based on NMR spectroscopy (Supplementary Fig. S1) and reverse-phase and chiral high-performance liquid-chromatography (HPLC) analyses.

QZ-Ala was obtained as a white solid with a 32% yield. ^13^C NMR (75 MHz, CDCl_3_) δ (p.p.m.): 178.1 (3C), 160.0 (3C), 149.7 (3C), 131.4 (3C), 50.3 (3C), 25.2 (3C). ^1^H NMR (300 MHz, CDCl_3_) δ (p.p.m.): 8.86 (s, 3 × 1H), 8.50 (d, *J* = 7.9 Hz, 3 × 1H), 5.67 (dq, *J* = 13.7, 6.8 Hz, 3 × 1H), 1.70 (d, *J* = 6.8 Hz, 3 × 3H). HRMS (ESI-TOF) calculated for C_18_H_19_N_6_O_3_Se_3_: (*M* + H)^+^, 606.9009; found, 606.9008.

The data for the characterization of QZ-Val have previously been reported (Aller *et al.*, 2009 ▶).

QZ-Leu was obtained as a white solid with a 45% yield. ^13^C NMR (75 MHz, CDCl_3_) δ (p.p.m.): 177.3 (3C), 160.1 (3C), 150.1 (3C), 130.9 (3C), 51.7 (3C), 47.9 (3C), 25.2 (3C), 23.0 (3C), 22.5 (3C). ^1^H NMR (300 MHz, CDCl_3_) δ (p.p.m.): 8.76 (s, 3 × 1 H), 8.25 (d, *J* = 9.6 Hz, 3 × 1H), 5.85–5.56 (m, 3 × 1H), 1.98–1.87 (m, 3 × 1H), 1.84–1.68 (m, 3 × 2H), 1.04 (d, *J* = 5.9 Hz, 3 × 3H), 1.00 (d, *J* = 6.1 Hz, 3 × 3H). HRMS (ESI-TOF) calculated for C_27_H_37_N_6_O_3_Se_3_: (*M* + H)^+^, 733.0417; found, 733.0418.

QZ-Phe was obtained as a white solid with a 40% yield. ^13^C NMR (75 MHz, CDCl_3_) δ (p.p.m.): 175.0 (3C), 160.1 (3C), 149.5 (3C), 136.3 (3 × 2C), 131.5 (3C), 130.0 (3 × 2C), 128.9 (3C), 127.5 (3C), 55.5 (3C), 44.0 (3C). ^1^H NMR (300 MHz, CDCl_3_) δ (p.p.m.): 8.72 (s, 3 × 1H), 8.48 (d, *J* = 8.5 Hz, 3 × 1H), 7.32–7.21 (m, 3 × 3H), 7.17–7.11 (m, 3 × 2H), 5.76 (td, *J* = 9.0, 4.8 Hz, 3 × 1H), 3.50 (dd, *J* = 13.1, 4.7 Hz, 3 × 1H), 3.04 (dd, *J* = 13.0, 9.4 Hz, 3 × 1H). HRMS (ESI-TOF) calculated for C_36_H_31_N_6_O_3_Se_3_: (*M* + H)^+^, 834.9948; found, 834.9949.

### 2.2. Measurement of ATPase activity   

The ATPase activity of P-gp was measured at 310 K using the ATP-regenerating system described by Vogel & Steinhart (1976 ▶) as modified by Urbatsch *et al.* (1995 ▶). Briefly, 1 µg P-gp was added to 100 µl 50 m*M* Tris–HCl pH 7.5 buffer containing 10 m*M* ATP, 12 m*M* MgCl_2_, 6 m*M* phosphoenolpyruvate, 1 m*M* NADH, 10 units of lactate dehydrogenase, 10 units of pyruvate kinase and test compounds over a range of concentrations. ATP hydrolysis was determined by the decrease in NADH absorbance at OD_340_ using a Filtermax F5 Multiplate Spectrophotometer. The ATPase activity was calculated using the equation ΔOD/(∊ × [protein] × time), where ΔOD is the change in absorbance and ∊ is the extinction coefficient of NADH. The concentration of purified P-gp was estimated by comparing the SDS–PAGE intensity of Coomassie-stained protein bands with known amounts of BSA. EC_50_ values were calculated using *GraphPad Prism* (GraphPad Software, San Diego, California, USA) using nonlinear regression (curve fit) from the entire concentration range.

### 2.3. Calcein-AM transport assay   

Calcein-AM (calcein acetoxymethyl ester) is a membrane-permeable P-gp substrate, while its free acid, calcein, hydrolyzed by endogeneous esterases, is trapped in the cytosol and exhibits strong fluorescence. It has commonly been used in fluorescence-based transport assays for P-gp in whole-cell systems (Al-Shawi & Senior, 1993 ▶). Chinese hamster ovary CR1R12 cells were cultured in the presence of 5 µg ml^−1^ colchicine to maintain P-gp overexpression. In general, ∼5 × 10^5^ CR1R12 cells per well were pretreated with test compounds in serially increasing concentrations at room temperature for 15 min; calcein-AM (0.25 µ*M*) was then added and incubated for an additional 15 min at room temperature while monitoring the fluorescence intensity (excitation at 485 nm, emission at 535 nm). 100% was the maximum fluorescence achieved by full inhibition of P-gp in CR1R12 cells. The means and standard deviations were obtained from quadruplet experiments.

### 2.4. Sensitization assay   

CR1R12 cells were grown in the presence of increasing concentrations of colchicine for 3 d at given concentrations of test compounds. Cell densities were determined using the sulforhodamine B colorimetric assay (Vichai & Kirtikara, 2006 ▶). 100% is defined as the growth in the absence of colchicine. Parental AUXB1 cells were included as controls.

### 2.5. Expression, purification, reductive methylation and crystallization of P-gp   

Gene-optimized mouse P-gp (*ABCB1a*; GenBank JF834158) was expressed in *Pichia pastoris* in 10 l cultures in a BioFlo 415 bioreactor (New Brunswick Scientific) and was induced by the slow addition of methanol (3.6 ml per hour per litre of culture volume) overnight as described previously (Aller *et al.*, 2009 ▶; Bai *et al.*, 2011 ▶). Cells were lysed at 287 MPa by a single pass through a Constant cell disrupter (TS Series, Constant Systems Inc.). Cellular debris was removed by centrifugation (12 500*g*, 20 min, 277 K) and crude membranes were prepared by centrifugation at 38 000*g* for 2–3 h at 277 K. P-gp was purified as described previously (Aller *et al.*, 2009 ▶) with modifications. Briefly, membranes containing P-gp were resuspended in cold buffer (100 m*M* NaCl, 15% glycerol, 20 m*M* Tris pH 8.0, 23.4 µ*M* leupeptin, 7 µ*M* E-64, 4 µ*M* chymostatin, 14.5 µ*M* pepstatin A, 1 m*M* PMSF, 25 m*M* benzamidine) and solubilized with 4.5% Triton X-100 for 1–2 h at 277 K. Centrifugation at 38 400*g* and 277 K for 30–60 min removed insoluble material, and the supernatant was applied onto Ni–NTA Superflow resin (Qiagen) using FPLC (ÄKTA, GE Life Sciences). The column was then washed with buffer consisting of 0.5 m*M* TCEP (Thermo Scientific), 0.04% sodium cholate (Sigma), 20 m*M* imidazole pH 8.0, 4.5% Triton X-100, 100 m*M* NaCl, 20 m*M* Tris–HCl pH 8.0, 14% glycerol. Resin-immobilized P-gp was buffer-exchanged into 20 m*M* HEPES pH 8.0, 0.2 m*M* TCEP, 100 m*M* NaCl, 20 m*M* imidazole, 0.04% sodium cholate, 0.065% β-DDM. P-gp was eluted with 20 m*M* HEPES pH 7.5, 100 m*M* NaCl, 0.2 m*M* TCEP, 0.04% sodium cholate, 200 m*M* imidazole pH 7.5, 0.065% β-DDM. The eluted protein was then diluted 1:10 with 20 m*M* HEPES pH 8.0, 100 m*M* NaCl, 0.2 m*M* TCEP, 0.04% sodium cholate, 0.065% β-DDM and rebound to Ni–NTA Superflow resin. This resin was washed with the buffer described above containing 20 m*M* imidazole and eluted with the buffer described above containing 200 m*M* imidazole. The protein was then concentrated (Centricon YM-50 or YM-100; Millipore), spun at 95 000 rev min^−1^ (TLA120.1 rotor) for 30–60 min at 277 K and subjected to size-exclusion chromatography (SEC; Superdex 200 16/60, GE Healthcare) at 277 K.

Following SEC, P-gp (at ∼1–2 mg ml^−1^) was subjected to reductive methylation (Rayment, 1997 ▶). Freshly made borane dimethylamine and formaldehyde were added to the protein solution to final concentrations of 50 and 100 m*M*, respectively, and then incubated for 2 h at 4°C. The reaction was quenched by the addition of ice-cold 25 m*M* glycine and was incubated for 30 min at 4°C. Methylated P-gp was then concentrated to 1 ml (Centricon YM-50 or YM-100; Millipore), diluted with 9 ml quench buffer (20 m*M* HEPES pH 7.5, 100 m*M* NaCl, 0.2 m*M* TCEP, 0.01% sodium cholate, 0.035% β-DDM) and this concentration/dilution step was repeated two times. In cases of cyclopeptide co-crystallizations, the appropriate compound was added from a 50 m*M* stock in 100% DMSO to a final concentration of 2 m*M* cyclopeptide in a 10 ml aliquot of P-gp at ∼1–2 mg ml^−1^ and incubated overnight. The following morning, excess cyclopeptide was removed by concentrating to ∼1 ml and dilution to ∼15 ml using quench buffer; this was repeated twice prior to concentrating for crystallization trials.

P-gp crystals were grown in 24-well Cryschem plates (Hampton Research) at a protein concentration of ∼10–12 mg ml^−1^ using 4 µl sitting drops at a 1:1 protein:mother liquor ratio using a well mother liquor consisting of 0.1 *M* HEPES pH 7–8, 50 m*M* lithium sulfate, 10 m*M* EDTA, 24–29.5%(*w*/*v*) PEG 600. Crystals were grown at 277 K; they typically appeared after 1–3 d and continued to grow to full size in approximately two weeks.

Collected crystals were first cryoprotected by soaking in 0.1 *M* HEPES at a pH identical to the crystal-growth condition, 50 m*M* lithium sulfate, 10 m*M* EDTA, 32% PEG 600. Crystals were typically ∼650 × 400 × 300 µm in size.

### 2.6. X-ray data collection, structure determination and refinement of P-gp cyclopeptide co-crystal structures   

X-ray diffraction data were collected at 100 K at either the Stanford Synchrotron Radiation Laboratory (SSRL; BL11-1) or the Canadian Light Source (CLS; 08ID-1). Fluorescence scans were taken on P-gp–cyclopeptide co-crystals to maximize the anomalous signal contribution from the incorporated selenium (Table 1 ▶). All diffraction data were processed with *MOSFLM* (Battye *et al.*, 2011 ▶) and reduced with *SCALA* (Evans, 2006 ▶) within the *CCP*4 suite of programs (Winn *et al.*, 2011 ▶). In the case of QZ-Ala, the data from three isomorphous crystals were scaled together to maximize the completeness (Table 1 ▶). The 3.4 Å resolution structure of P-gp was initially solved by molecular replacement (MR) with *Phaser* (McCoy *et al.*, 2007 ▶) using a previously determined P-gp structure (PDB entry 4ksc; Ward *et al.*, 2013 ▶) as a search model with no modifications. Commensurate with the improved resolution, the new electron-density features guided adjustments of our model when compared with the same more ‘open’ crystal form that we reported in 2013 (Ward *et al.*, 2013 ▶) and are summarized in Supplementary Fig. S2. Residues 30–32 were located in the electron density, and resulted in a subsequent shift in the registration of residues in the first helix (residues 30–43) preceding TM1. Amendments were made to the topology of intracellular helix 1 (IH1; residues 154–168), extracellular loop 3 (ECL3; residues 318–338) and a portion of TM6 leading into the first NBD (residues 358–387). Within NBD1, residues 398–404, 424–427, 520–526 and 597–602 were rebuilt. Elbow helix 2 (EH2) was rebuilt from residues 689 to 708. A registry issue was amended from ECL4 (residue 738) to TM8 (residue 760) and another that constitutes segments of TM9, ECL5 and the beginning of TM10 (residues 826–855). The topology of IH3 was adjusted (residues 795–806), as was ECL6 (residues 961–967) and a portion of TM12 (residues 972–984). Further modifications were made in the region leading into and contributing to NBD2 (residues 1010–1028, 1042–1047, 1129–1137 and 1165–1172). Residues 1272 and 1273 were also located in the electron-density maps at the C-terminus. As for all structures of P-gp determined to date, the ‘linker’ region (residues 627–688) was not located in the electron density. Many of the structural adjustments are in general agreement with the recent corrections (Li *et al.*, 2014 ▶) made to the model of the more ‘closed’ conformation of P-gp first reported in 2009 (Aller *et al.*, 2009 ▶). During the refinement process, the model underwent rigid-body and restrained positional refinement, with H atoms applied in their riding positions, using *phenix.refine* (Afonine *et al.*, 2012 ▶) against a maximum-likelihood target function with grouped *B* factors, secondary-structure restraints, reference-model restraints and TLS. Rounds of refinement were interspersed with manual inspection and correction against σ_A_-weighted electron-density maps in *Coot* (Emsley *et al.*, 2010 ▶) and improvements to model geometry and stereochemistry were monitored using *MolProbity* (Chen *et al.*, 2010 ▶). Subsequent cyclopeptide co-crystal structures were solved by either MR or rigid-body refinement using the refined 3.4 Å resolution model with residues from TM4 (218–243) and EH2 (689–694) removed to avoid biasing their placement within the electron-density maps. These structures were then refined in a similar fashion to the 3.4 Å resolution structure with an additional round of positional refinement with ligand *B* factors set to the Wilson *B* value. Ligand description dictionaries were determined using *phenix.elbow* (Adams *et al.*, 2010 ▶) and the crystallographic positions of the incorporated seleniums were validated using anomalous scattering methods. The refined structures were judged to have excellent geometry as determined by *MolProbity* (Chen *et al.*, 2010 ▶). The resulting refinement statistics are listed in Table 1 ▶.

## 3. Results   

### 3.1. Rational engineering and functional characterization of cyclopeptide P-gp ligands   

**Figure 1:**
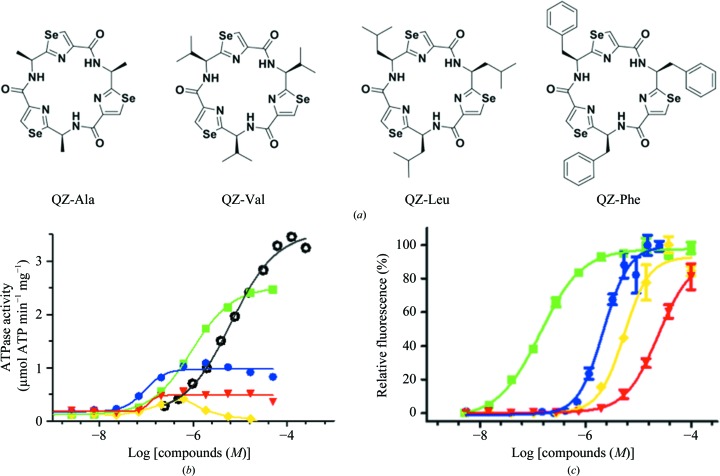
Structure and function of selenium-labelled homotrimeric cyclopeptides. (*a*) The chemical structures of the cyclopeptide series bear an identical backbone but with side chains systematically increasing in size and hydrophobicity (from left to right). (*b*) Stimulation of the basal ATPase activity of purified P-gp. QZ-Ala (green) conferred the highest degree of stimulation (∼16-fold) relative to the basal activity, with an EC_50_ value of 0.92 µ*M*, followed by QZ-Val (blue; ∼7-fold). Data for verapamil (black), QZ-Leu (yellow) and QZ-Phe (red) are shown. (*c*) Inhibition of calcein-AM transport in P-gp-overexpressing CR1R12 cells. The same color scheme is applied for each compound as in (*b*) and the data were fitted using the Hill equation. The mean and SD of triple and quadruplet experiments are shown in (*b*) and (*c*), respectively.

Previous functional studies have identified at least four, and potentially up to seven, sometimes overlapping binding sites for substrates and inhibitors in the greasy, polyspecific binding cavity of P-gp (Shapiro & Ling, 1997 ▶; Martin *et al.*, 2000 ▶). To probe this phenomenon, the cyclic peptide QZ59-SSS (here named QZ-Val), previously reported in a co-crystal structure of mouse P-gp/Mdr1a (Aller *et al.*, 2009 ▶), was taken as a base to engineer a series of selenium-labeled homotrimeric cyclopeptides (Fig. 1 ▶ *a*). The *R* groups of this series were systematically varied to generate alanine-, valine-, leucine- and phenylalanine-derived compounds (QZ-Ala, QZ-Val, QZ-Leu and QZ-Phe, respectively) of increasing *R*-group size and hydrophobicity.

We measured the effect of each ligand on the basal level of ATP hydrolysis of purified P-gp. Our results revealed that the smaller compounds were more stimulatory and, most notably, the smallest ligand, QZ-Ala, potently stimulated ATP hydrolysis in a dose-dependent manner at the tested concentrations, similar to verapamil (Fig. 1 ▶ *b*). Consistent with these substrate-like interactions, P-gp mediated mild resistance to QZ-Ala in cells (Supplementary Fig. S3). These data suggest that the binding of QZ-Ala and, to a lesser degree, QZ-Val at the TMDs generates a signal to the NBDs, accelerating nucleotide hydrolysis. We also characterized the ligands using P-gp-overexpressing CR1R12 cells. A pattern emerged in P-gp-mediated calcein-AM transport out of cells, whereby the potency of the ligand to inhibit export was inversely related to *R*-group size (Fig. 1 ▶ *c*). The smaller QZ-Ala inhibited calcein-AM export (IC_50_ = 140 n*M*) best compared with QZ-Val (IC_50_ = 1.7 µ*M*), QZ-Leu (IC_50_ = 5.4 µ*M*) and QZ-Phe (IC_50_ = 24 µ*M*) (Fig. 1 ▶ *c*). Fitting the data to the Hill equation gave Hill coefficients of >1 in each case, suggesting positive cooperativity for their binding to at least two sites. All four compounds also prevented P-gp-mediated export of the anticancer drug colchicine and sensitized CR1R12 cells in a dose-dependent manner (Supplementary Fig. S4).

### 3.2. Structures of P-gp in complex with cyclopeptide ligands   

**Figure 2:**
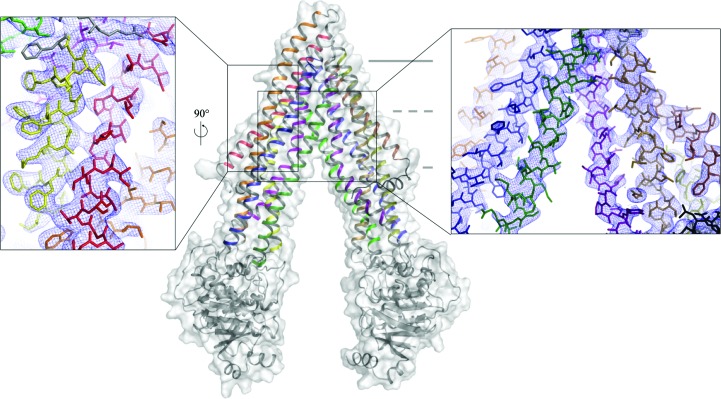
Overview of mouse P-gp at 3.4 Å resolution. Magnified insets at different orientations are shown with the resulting 2*mF*_o_ − *DF*_c_ electron density (where *m* is the figure of merit and *D* is the σ_A_ weighting factor) contoured at 1σ; individual transmembrane helices are shown in different colors. TM1 is in red, TM2 is in orange, TM3 is in yellow, TM4 is in light green, TM5 is in sky blue, TM6 is in pink, TM7 is in dark brown, TM8 is in olive, TM9 is in light brown, TM10 is in forest, TM11 is in dark blue and TM12 in deep purple.

Prior to determining P-gp–ligand co-crystal structures, an improved structure of apo mouse P-gp/Mdr1a was determined to 3.4 Å resolution. To date, this model is the highest resolution structure reported for a mammalian ABCB1/MDR1-type transporter (87% identity to human P-gp; Fig. 2 ▶, Table 1 ▶). This model is similar to those recently described (Ward *et al.*, 2013 ▶) and differs significantly from the original reported in 2009 (Aller *et al.*, 2009 ▶). The increase in resolution has also facilitated electron-density map-driven improvements to the model (see §2 *Materials and methods*; Fig. 2 ▶, Table 1 ▶), resulting in better refinement statistics.

**Figure 3:**
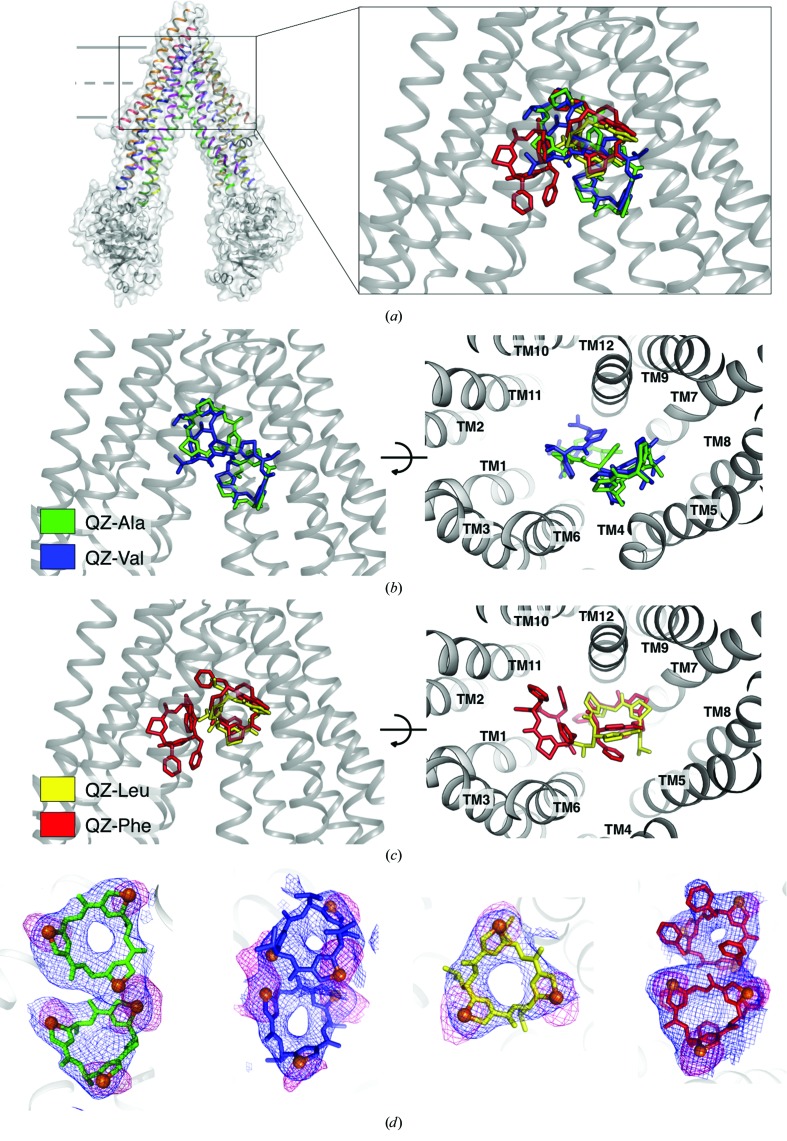
Overview of P-gp–cyclopeptide co-crystal structures. (*a*) Superposition of homotrimeric cyclopeptide compounds bound to P-gp, showing their relative location and orientation in the substrate-binding pocket. (*b*) Two orientations of subset A ligands (QZ-Ala and QZ-Val; displayed as sticks) bound in the substrate-binding pocket of P-gp. QZ-Ala is shown in green and QZ-Val in blue. (*c*) Two orientations of subset B ligands (QZ-Leu and QZ-Phe) bound in the substrate-binding pocket of P-gp. QZ-Leu is shown in yellow and QZ-Phe in red. (*d*) Close-up view of ligands, colored as in (*b*) and (*c*), with the resulting 2*mF*_o_ − *DF*_c_ electron density in blue (contour level of 1.0σ) and anomalous difference density peaks in pink (contour levels of 3.0σ for QZ-Ala, QZ-Va and Q-Phe and 4.0σ for QZ-Leu) for the Se atoms (orange spheres).

**Figure 4:**
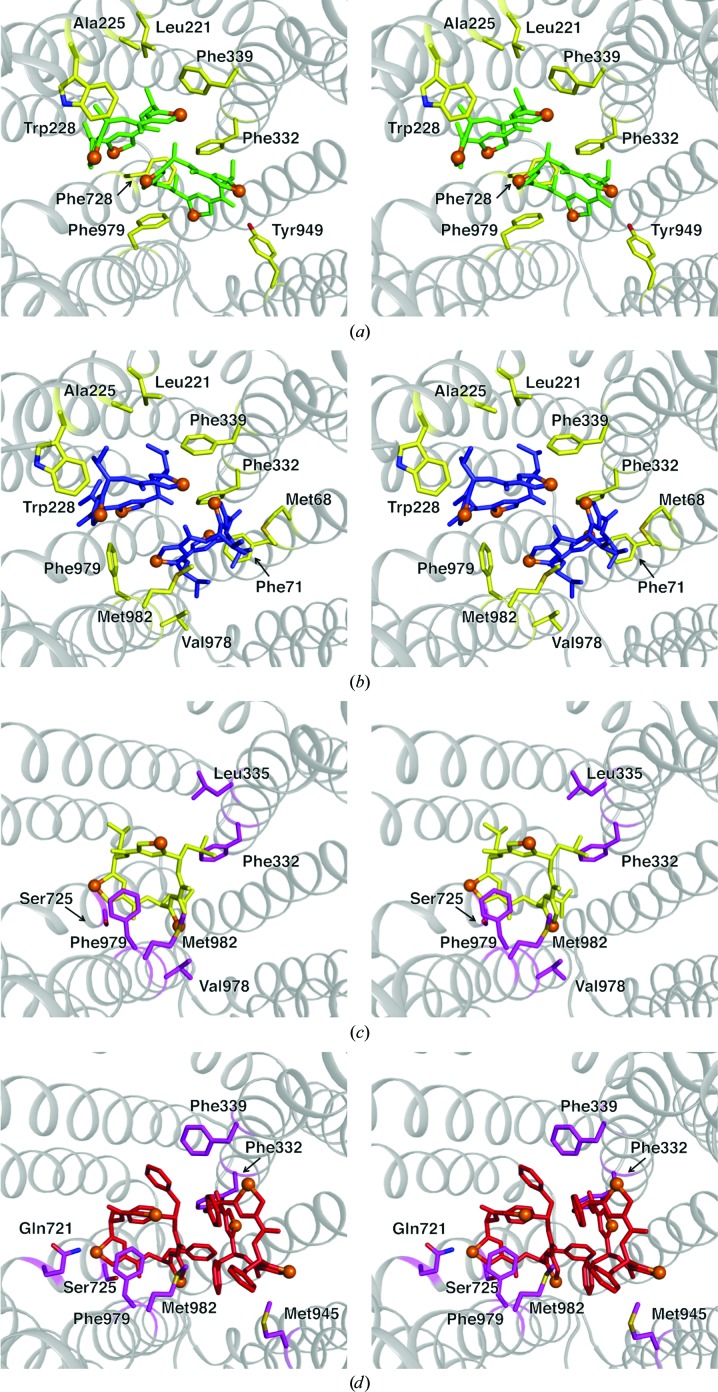
Key P-gp residues involved in binding homotrimeric cyclopeptides. Stereoviews of the binding pocket are shown perpendicular to the membrane and viewed from the cytosol. P-gp residues are shown as sticks, with those involved in binding subset A ligands colored yellow and those involved in binding subset B ligands colored magenta. The ligand coloring is consistent with that shown in Fig. 3 ▶; QZ-Ala, green; QZ-Val, blue; QZ-Leu, yellow; QZ-Phe, red.

To interrogate *R*-group-dependent variations in ligand binding, co-crystal structures of P-gp with each ligand were determined (Figs. 3 ▶ *a*, 3 ▶ *b*, 3 ▶ *c* and 4 ▶). Ligands were placed using difference electron densities and strong anomalous scattering from the three triangulated Se atoms (Fig. 3 ▶ *d*). All ligands bound to P-gp (Fig. 3 ▶ *d*), and the majority of the coordinating side chains, are well resolved in the electron-density maps. The ligands make a number of interactions with binding-pocket residues (Fig. 4 ▶), burying a high proportion of the solvent-accessible surface area of the ligand (Supplementary Table S1).

## 4. Discussion   

### 4.1. Comparison of cyclopeptide ligands in complex with P-gp   

Our P-gp–ligand co-crystal structures demonstrate the wide range of possible binding modes for similar substrates (Fig. 3 ▶ *a*). Within the binding pocket, our structures group the four ligands into two subsets that correlate with size and hydrophobicity (Figs. 3 ▶ *b*, 3 ▶ *c* and 4 ▶). The smaller ligands QZ-Ala and QZ-Val (subset A) share an upper and lower binding site (Figs. 3 ▶ *b* and 4 ▶), while the larger and more hydrophobic ligands QZ-Leu and QZ-Phe (subset B) share a different upper binding site, with QZ-Phe also binding to a second, unique lower site (Figs. 3 ▶ *c* and 4 ▶). P-gp consists of two pseudo-symmetric halves, each containing six transmembrane (TM) helix bundles. Viewed from the plane of the membrane, the ligands bound in the upper sites of subsets A and B are wedged in the apex of the cavity, engaging TM helices from both pseudo-halves (Figs. 3 ▶ *b* and 3 ▶ *c*). In contrast, the ligands in the lower sites interact with distinct pseudo-halves of P-gp that are mutually exclusive to each ligand subset (Figs. 3 ▶ *b*, 3 ▶ *c* and 4 ▶).

### 4.2. Movement of TM4 upon ligand binding   

**Figure 5:**
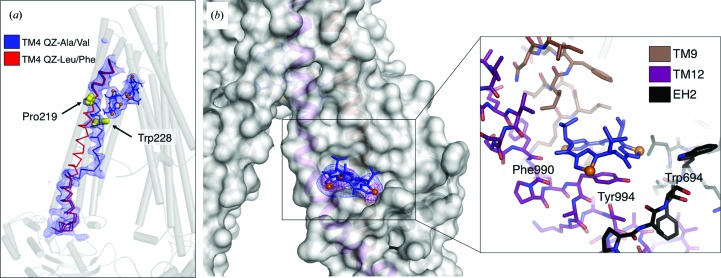
Overview of the EH2 ligand-binding site and the ligand-induced movement of TM4. (*a*) The kinking of TM4 in response to subset A ligands is shown (blue ribbon) in comparison to its ‘straight’ topology in the subset B co-crystal structures (red ribbon). The position of QZ-Val ligands and the resulting 2*mF*_o_ − *DF*_c_ electron density (contoured at 1.0σ) are displayed. Residues Pro219 and Trp228 are shown as yellow spheres. (*b*) QZ-Val bound at the EH2 site with P-gp rendered as a molecular-surface representation. The resulting 2*mF*_o_ − *DF*_c_ electron density for the ligand is shown at a contour level of 1.0σ and the resulting anomalous difference peaks for the Se atoms (orange spheres) are shown at 3.5σ. The close-up inset view depicts the surrounding TM9 and TM12 as brown and purple sticks, respectively, and EH2 as black sticks. Residues in close vicinity to the ligand (Trp694, Phe990 and Tyr994) are labeled.

In previous structures of apo mouse P-gp, and also for ligands pertaining to subset B, TM4 adopts a mostly straight-helical conformation characterized by weaker electron density, indicative of regional flexibility (Ward *et al.*, 2013 ▶). Subset A ligands not only revealed an ordered TM4, but a large conformational change (of up to 11 Å when comparing corresponding C^α^ positions; Fig. 5 ▶ *a*). This structural kink is not likely to be a consequence of lattice contacts as the crystal form is the same (Table 1 ▶). Thus, we must conclude that the binding of subset A ligands induces these changes in TM4. This ligand binding-induced kinking of TM4 begins at Pro219 before returning to the apo wild-type topology at Tyr243 in the ball-and-socket region (Loo *et al.*, 2013 ▶) close to NBD2.

The observed movement of TM4 upon substrate/ligand binding may have significant biological implications. TM4 and TM6 comprise an intramembranous portal for substrate entry to the binding cavity, and a conformational change in these helices may influence the entry and the binding of ligands (Loo & Clarke, 1994 ▶, 2005 ▶; Woebking *et al.*, 2008 ▶). In the recent structure of *Cyanidioschyzon merolae* P-gp, a portion of TM4 in the wild-type protein was inherently disordered (Kodan *et al.*, 2014 ▶). Mutations in this region not only resulted in a well ordered, straight-helical conformation, but also functionally disrupted substrate transport, suggesting a role for TM4 in facilitating substrate entry and/or binding (Kodan *et al.*, 2014 ▶). For co-crystal structures pertaining to subset A ligands, the movement of TM4 brings residues 221–228 closer to the bound ligands in the lower binding sites, fostering an intermolecular interaction with Trp228, a residue that has been implicated in steroid binding to P-gp (Gruol *et al.*, 2002 ▶). We propose that the movement of TM4 upon binding subset A compounds provides a structural glimpse of the induced-fit model of drug binding proposed for P-gp nearly a decade ago (Loo *et al.*, 2003*b* ▶).

Several ligands stimulate the basal rate of ATP hydrolysis of P-gp by severalfold (Al-Shawi & Senior, 1993 ▶; Ambudkar *et al.*, 1992 ▶; Scarborough, 1995 ▶; Fig. 1 ▶ *b*). However, the mechanisms coupling ligand binding in the TMDs to increased ATP turnover at the NBDs are not fully understood. Cross-linking and FRET studies on P-gp and the bacterial homolog MsbA have suggested that ligand binding induces closure of the NBDs in the presence of nucleotides, leading to the increase in catalysis (Szabo *et al.*, 1998 ▶; Eckford & Sharom, 2008 ▶; Liu & Sharom, 1996 ▶; Loo *et al.*, 2003*a* ▶; Siarheyeva & Sharom, 2009 ▶; Wang *et al.*, 1998 ▶; Scarborough, 1995 ▶; Doshi & van Veen, 2013 ▶). Specifically, conformational changes in TM4 have been linked to this TMD–NBD coupling (Doshi & van Veen, 2013 ▶), consistent with our models. In our structures, ligands that function more as activators of ATPase (Fig. 1 ▶ *b*; QZ-Ala and QZ-Val; subset A) kink TM4, while those that function more as inhibitors of ATPase activity (QZ-Ile and QZ-Phe; subset B) maintain straight TM4 helices as also observed in the apo structure. Here, we only demonstrate two distinct changes in TM4 (kinked or straight helical) caused by two different subsets of ligands. Other ligand-binding sites in the TMD are possible, resulting in corresponding structural changes for TM4 or perhaps other TM helices extending to the NBDs. Variations in the degree of structural kink of TM helices, for example, are likely for other compounds.

In this study, we cannot resolve long-range structural changes of the intracellular helix (residues 242–256) extending to NBD2 that would provide further insights regarding the exact nature of any potential coupling mechanism between TM4 and NBD2. Several possibilities could explain this observation. For example, the structural changes in TM4 extending to NBD2 might only occur as the transporter goes from a wide-open to a closed inward-facing conformation where the NBDs begin to contact. These changes may also necessitate the presence of ATP, which is absent in these structures. Another possibility is that these structural changes may be too small to resolve using our current data. If so, computational studies using these models incorporating other biochemically derived restraints could be very valuable for understanding how substrate-stimulated ATP hydrolysis in P-gp may be initiated. Taken together with other complementary studies (Loo *et al.*, 2003*a* ▶; Doshi & van Veen, 2013 ▶), our structures provide a starting molecular-structural framework for a ligand induced-fit mechanism transmitting structural changes from the TMDs to the NBDs.

### 4.3. Ligand-binding site at the membrane interface   

Previous biochemical experiments have led to the proposal that P-gp extrudes drugs from the inner leaflet of the plasma membrane, functioning as a so-called ‘hydrophobic vacuum cleaner’ (Raviv *et al.*, 1990 ▶; de Graaf *et al.*, 1996 ▶; Bolhuis *et al.*, 1996 ▶). In our QZ-Val co-crystal structure, we observed an additional binding site on the exterior of P-gp bounded by residues from TM9, TM12 and EH2 (Fig. 5 ▶ *b*). The site faces away from the transporter, but lies close to the predicted membrane–water interface and intramembranous substrate-entry portal. Drug binding near the EH has been reported using electron paramagnetic resonance on a bacterial P-gp homolog that transports lipids (Smriti *et al.*, 2009 ▶). These data lend strong credence to the proposal of an initial lower-affinity ‘ON-site’ for a ligand near the inner leaflet of the lipid bilayer preceding the higher-affinity ‘ON-site(s)’ within the central binding cavity (Dey *et al.*, 1997 ▶; Al-Shawi & Omote, 2005 ▶).

## 5. Conclusions   

Understanding drug–transporter interactions is indispensable for engineering drugs to inhibit or evade P-gp. The findings presented here complement and extend the related X-ray structures (Hohl *et al.*, 2012 ▶; Ward *et al.*, 2007 ▶, 2013 ▶; Shintre *et al.*, 2013 ▶; Kodan *et al.*, 2014 ▶; Jin *et al.*, 2012 ▶; Aller *et al.*, 2009 ▶), as well as many biochemical/biophysical studies (Juliano & Ling, 1976 ▶; Al-Shawi & Omote, 2005 ▶). We have conducted the first detailed structure–activity relationship (SAR) study on how altering the size and hydrophobicity of the *R* groups in a known P-gp ligand can impact its interactions with this clinically important protein. The mechanistic concepts delivered through this structural study include (i) ligand entry *via* the elbow helix binding site, (ii) distinct and shared binding modes and (iii) ligand binding-induced fit that could cause transmission coupled to ATP catalysis. These structural observations provide an excellent basis to biochemically and computationally test hypotheses in further studies on this transporter.

## 6. Related literature   

The following reference is cited in the Supporting Information for this paper: Krissinel & Henrick (2007 ▶).

## Supplementary Material

PDB reference: P-glycoprotein, 4q9h

PDB reference: cocrystallized with QZ-Ala, 4q9i

PDB reference: cocrystallized with QZ-Val, 4q9j

PDB reference: cocrystallized with QZ-Leu, 4q9k

PDB reference: cocrystallized with QZ-Phe, 4q9l

Supporting Information.. DOI: 10.1107/S1399004715000978/cb5079sup1.pdf

## Figures and Tables

**Table 1:** Data-collection and refinement statistics Values in parentheses are for the highest resolution shell.

	Native	QZ-Ala	QZ-Val	QZ-Leu	QZ-Phe
Data collection					
Wavelength ()	0.9795	0.9796	0.979	0.979	0.980
Beamline	11-1, SSRL	08ID, CLS	08ID, CLS	11-1, SSRL	11-1, SSRL
Space group	*P*2_1_2_1_2_1_	*P*2_1_2_1_2_1_	*P*2_1_2_1_2_1_	*P*2_1_2_1_2_1_	*P*2_1_2_1_2_1_
Unit-cell parameters					
*a* ()	88.0	85.6	88.7	91.3	86.3
*b* ()	139.2	138.4	138.7	138.5	138.4
*c* ()	186.0	183.7	190.0	195.6	185.0
= = ()	90	90	90	90	90
Resolution range ()	86.33.4 (3.63.4)	92.23.8 (4.03.8)	88.73.6 (3.83.6)	113.13.8 (4.03.8)	86.33.8 (4.03.8)
No. of crystals	1	3	1	1	1
*R*_merge_ (%)	8.4 (73.0)	9.8 (68.4)	7.9 (64.9)	5.2 (73.0)	5.8 (69.6)
*R*_p.i.m._ (%)	4.4 (38.3)	3.4 (25.8)	4.1 (35.0)	3.5 (46.4)	3.1 (36.6)
Observed reflections	150254	198281	120622	73344	97970
Unique reflections	31984	22299	26969	23166	22385
Mean *I*/(*I*)	8.1 (1.8)	11.9 (2.8)	8.4 (2.0)	9.8 (1.7)	10.9 (2.2)
Completeness (%)	99.3 (100)	99.6 (99.9)	97.4 (98.5)	92.4 (95.8)	99.4 (100)
Multiplicity	4.7 (4.6)	8.9 (7.7)	4.5 (4.3)	3.2 (3.2)	4.4 (4.4)
Refinement					
Resolution range ()	77.43.4 (3.53.4)	91.83.8 (3.93.8)	78.43.6 (3.73.6)	49.63.8 (3.93.8)	76.93.8 (3.93.8)
Reflections in working set	31868	22236	26927	22913	22339
Reflections in test set (%)	5.1	5.1	5.1	5.1	5.1
*R*_work_/*R*_free_ (%)	26.0/29.1	26.3/29.5	26.1/28.2	29.2/32.1	25.9/29.3
R.m.s deviations					
Bond lengths ()	0.007	0.004	0.004	0.004	0.004
Bond angles ()	1.026	0.765	0.801	0.758	0.755
*B* (^2^)	99.3	156.3	160.8	182.5	177.1
Ramachandran statistics					
Outliers (%)	0	0	0	0	0
Favoured (%)	95.9	95.7	95.3	95.3	95.7
Rotamer outliers (%)	1.0	0.3	0.5	0.4	0.2
C deviations	0	0	0	0	0
