# Supplementary material for: Snapshots of ligand entry, malleable binding and induced helical movement in P-glycoprotein
Source: Acta Crystallogr D Biol Crystallogr. 2015 Feb 26;71(Pt 3):732–41. doi: 10.1107/S1399004715000978 (PMC4356375; doi:10.1107/S1399004715000978)
Supplement: Supplementary file 1 [file d-71-00732-sup1.pdf]

BIOLOGICAL
CRYSTALLOGRAPHY

Volume 71 (2015)

Supporting information for article:

Snapshots of ligand entry, malleable binding and induced helical movement in P-glycoprotein

Paul Szewczyk, Houchao Tao, Aaron P. McGrath, Mark Villaluz, Steven D. Rees, Sung Chang Lee, Rupak Doshi, Ina L. Urbatsch, Qinghai Zhang and Geoffrey Chang

Table S1 Solvent accessible surface area of cyclopeptide ligands buried in the pocket of P-gp as calculated by PISA(Krissinel & Henrick, 2007)

	Accessible Surface Area (Å²)	Buried Surface Area (Å²)
QZ-Ala Upper	628.7	451.6
QZ-Ala Lower	623.3	409.1
QZ-Val Upper	723.1	513.6
QZ-Val Lower	690.5	475.4
QZ-Val Outer	723.9	426.7
QZ-Leu Upper	824.1	577.5
QZ-Phe Upper	760.4	577.2
QZ-Phe Lower	783.9	474.8

Figure S1 NMR spectra of QZ-Ala, QZ-Leu and QZ-Phe. ^{13}C (top) and ^{1}H (bottom) spectra are shown for each compound.

Figure S2 Structure based alignment. Two views of P-gp depicting C_α position shifts between the new (P-gp native at 3.4 Å, reported here) and previously published model (pdb code: 4KSB). Changes are depicted by color with red indicating the largest shift in C_α position (8.35 Å). Alignments and pairwise C_α RMSD calculations were calculated using the python module 'ColorByRMSD' within the PyMOL Molecular Graphics System (Schrödinger, LLC)

Figure S3 Cytotoxicity of the homotrimeric cyclopeptide compounds to P-gp-over-expressing CR1R12, and parental control AuxB1, cells using Calcein-AM transport assay. P-gp mediates mild resistance to QZ-Ala. For QZ-Phe, full growth of inhibition was not observed due to limited solubility of this compound at high concentrations. The mean and SD of quadruplet experiments are shown; data were fit using the Hill equation. QZ-Ala, QZ-Val, QZ-Leu and QZ-Phe are drawn in green, blue, yellow and red curves, respectively. The concentrations are plotted in log scale.

Figure S4 Sensitization of CR1R12 cells to the anti-cancer drug colchicine by homotrimeric cyclopeptides. Relative to parental control AUXB1 cells, P-gp-overexpressing CR1R12 cells are ~200 times more resistant to the anticancer drug colchicine (gray lines in all panes). Their 50% growth inhibition (GI_{50}) values were 0.14 µM and 27 µM respectively. Presence of (a) QZ-Ala, green, (b) QZ-Val, blue, and (d) QZ-Phe, red, in the growth media sensitized the CR1R12 cells to colchicine in a concentration-dependent manner with full reversal of P-gp mediated multidrug resistance seen at 2 µM; this concentration gave essentially the same GI_{50} value as seen in AuxB1 cells. Presence of increasing concentrations of (c) QZ-Leu, yellow, also sensitized CR1R12 cells to colchicine, where full reversal of multidrug resistance was not observed because this compound became cytotoxic at higher concentrations. Shown are representative data from three independent experiments. The concentrations are plotted in log scale.

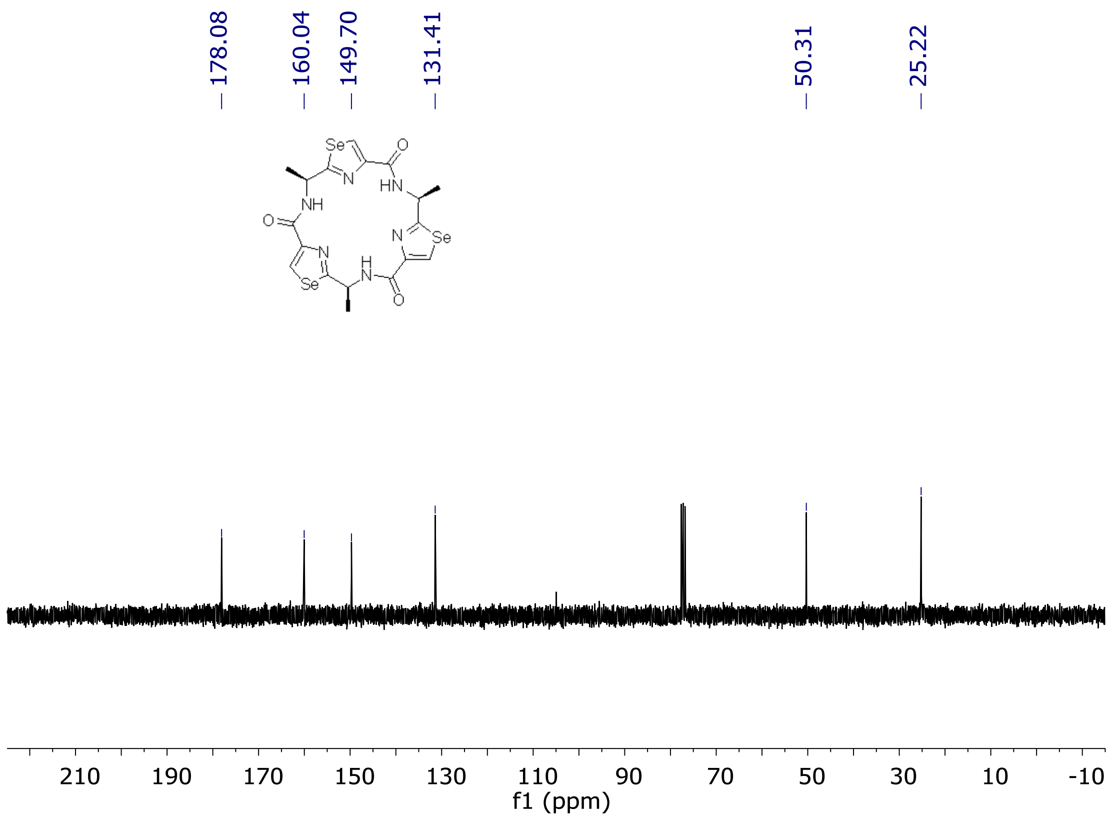
178.08
160.04
149.70
131.41
50.31
25.22
Se
N
O
HN
NH
O
N
Se
N
H
N
Se
O
210 190 170 150 130 110 90 70 50 30 10 -10
f1 (ppm)

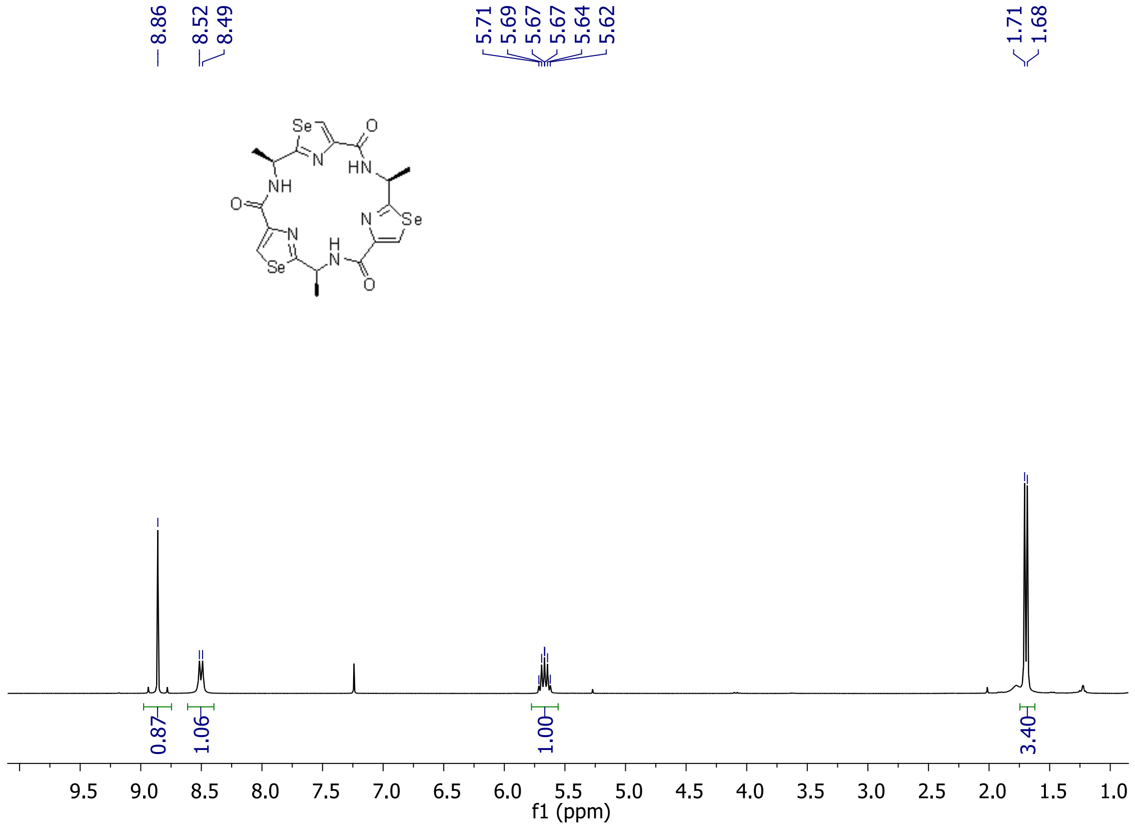
8.86
8.52
8.49
5.71
5.69
5.67
5.67
5.64
5.62
1.71
1.68
Se
N
O
HN
NH
O
N
Se
N
H
N
Se
O
0.87
1.06
1.00
3.40
9.5 9.0 8.5 8.0 7.5 7.0 6.5 6.0 5.5 5.0 4.5 4.0 3.5 3.0 2.5 2.0 1.5 1.0
f1 (ppm)

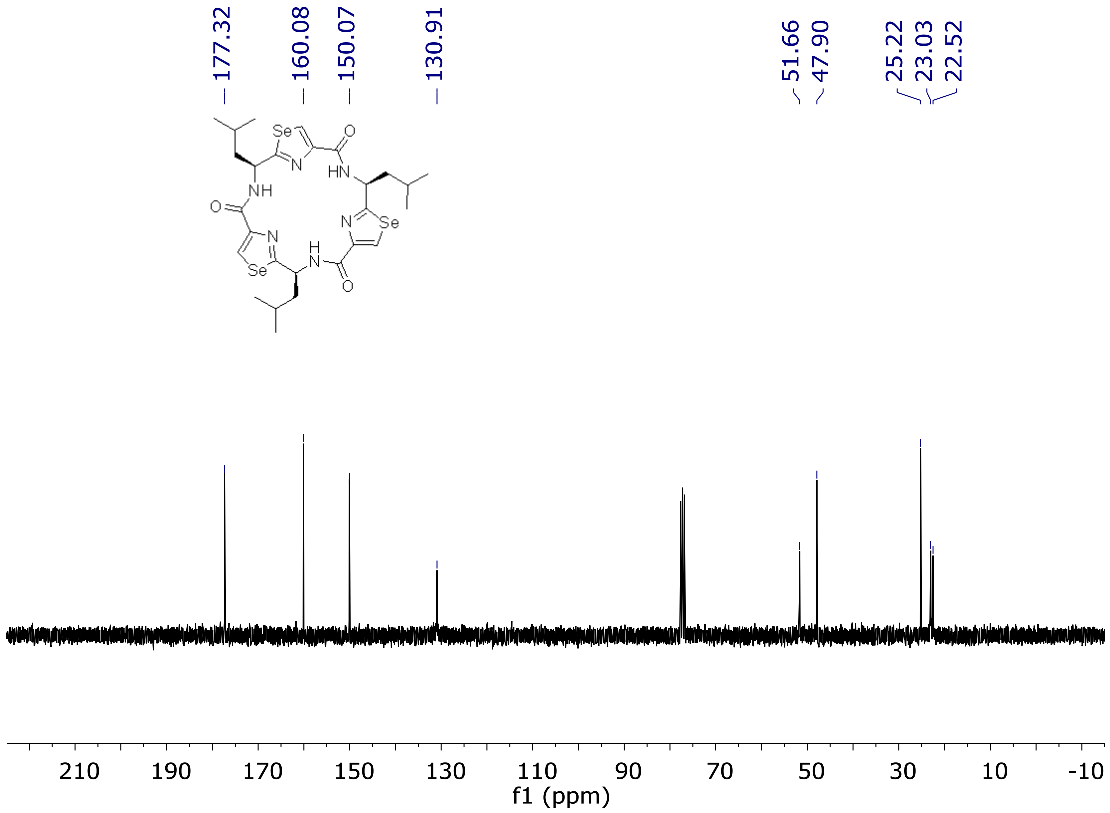
177.32
160.08
150.07
130.91
51.66
47.90
25.22
23.03
22.52
210 190 170 150 130 110 90 70 50 30 10 -10
f1 (ppm)

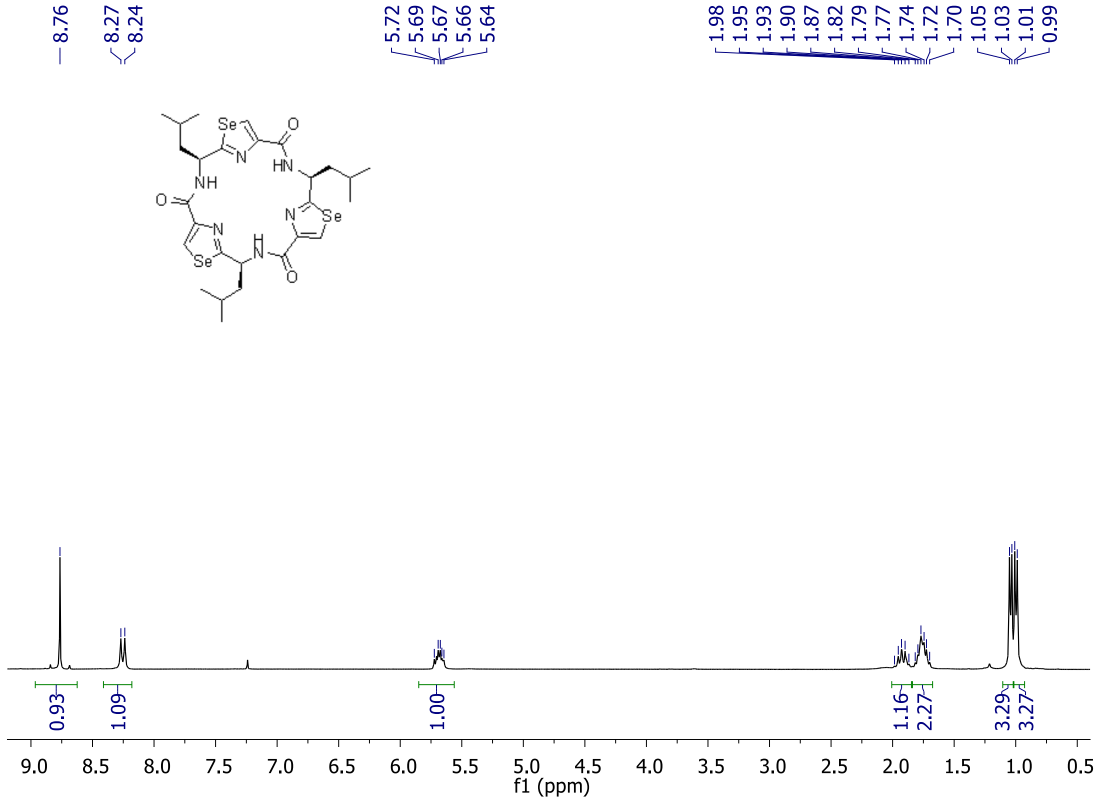
8.76
8.27
8.24
5.72
5.69
5.67
5.66
5.64
1.98
1.95
1.93
1.90
1.87
1.82
1.79
1.77
1.74
1.72
1.70
1.05
1.03
1.01
0.99
0.93
1.09
1.00
1.16
2.27
3.29
3.27
9.0 8.5 8.0 7.5 7.0 6.5 6.0 5.5 5.0 4.5 4.0 3.5 3.0 2.5 2.0 1.5 1.0 0.5
f1 (ppm)

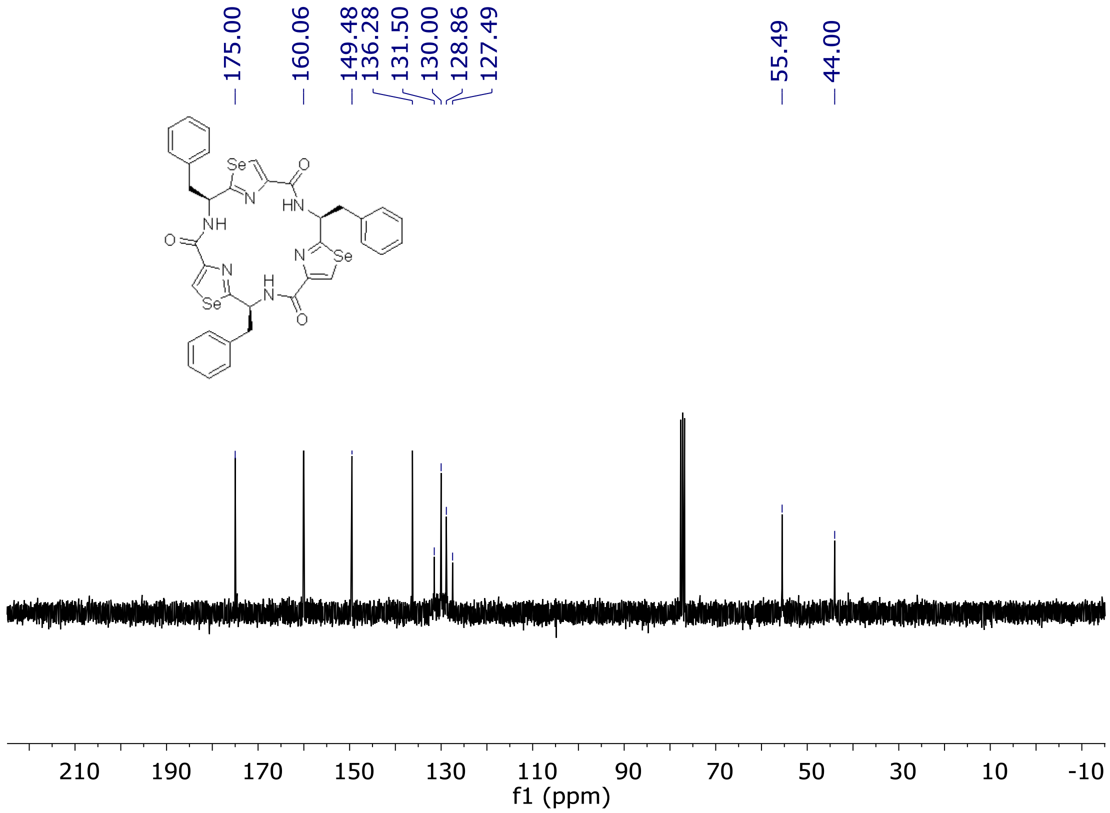

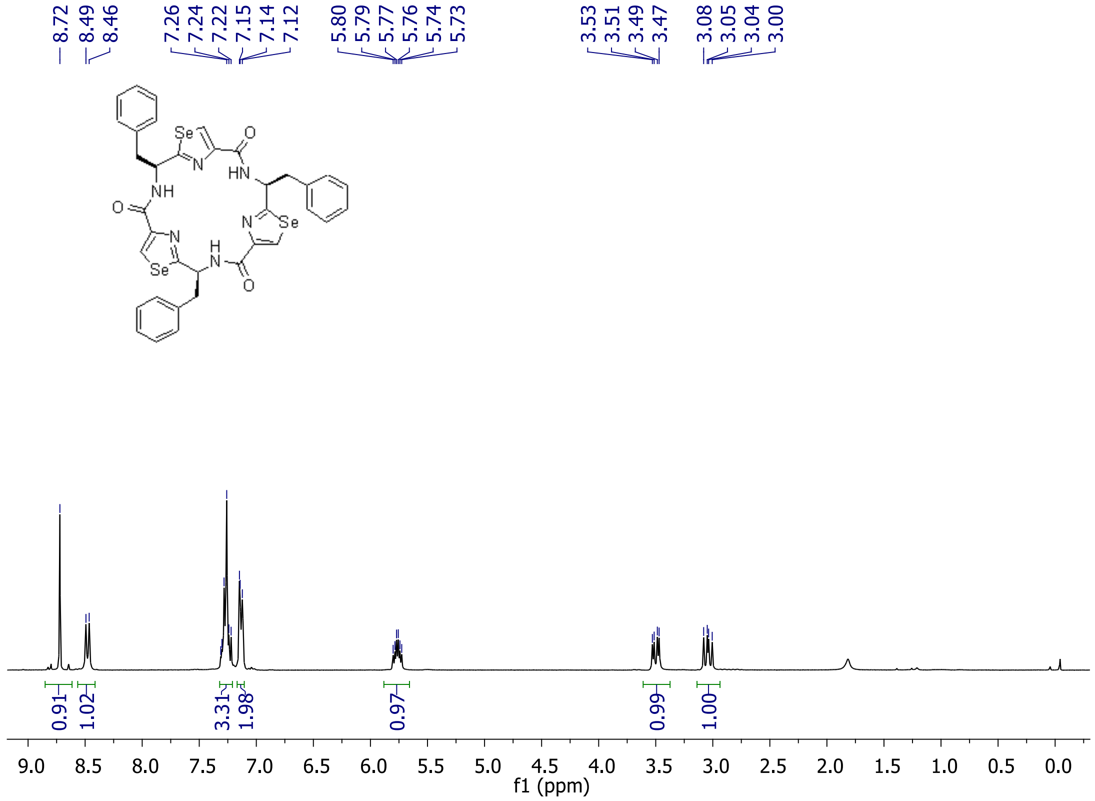

Supplementary Figure S1

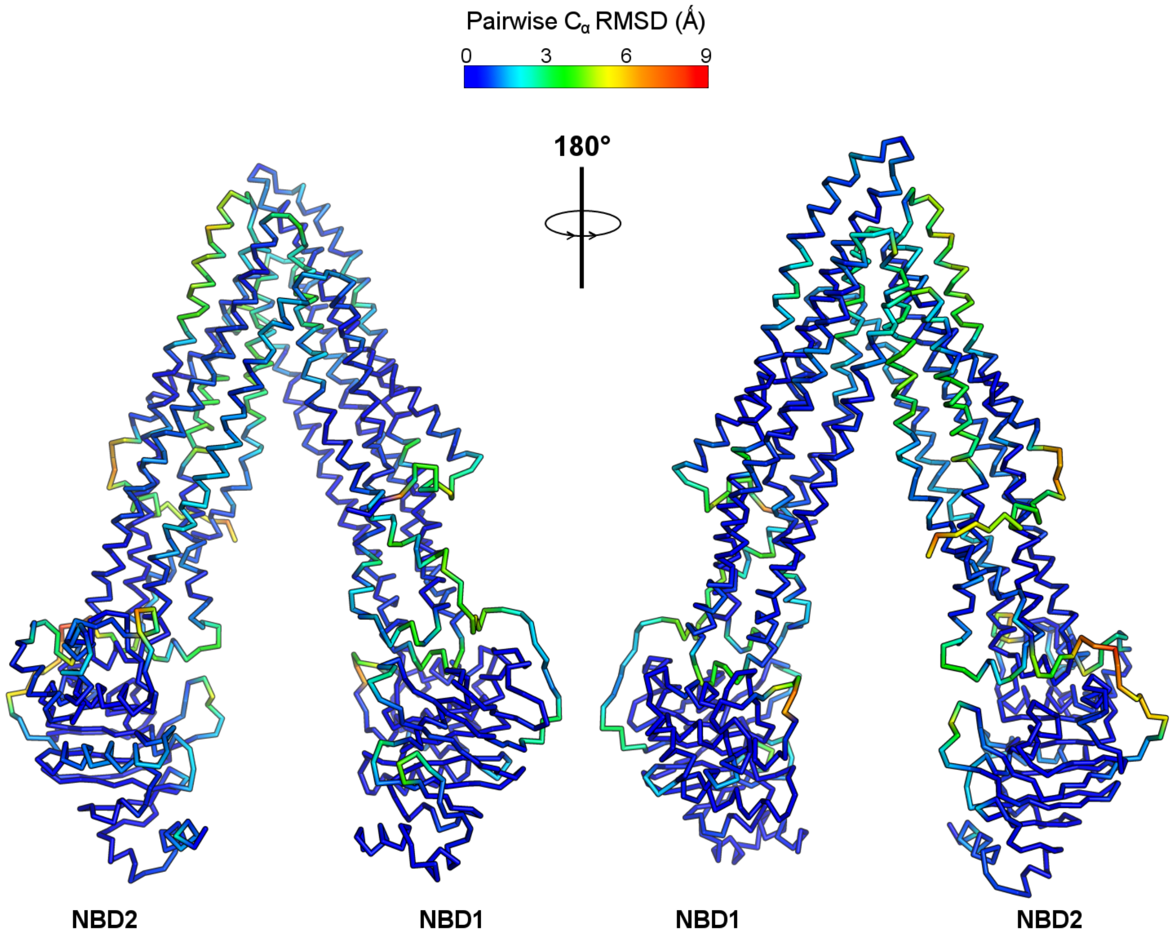

Supplementary Figure S2

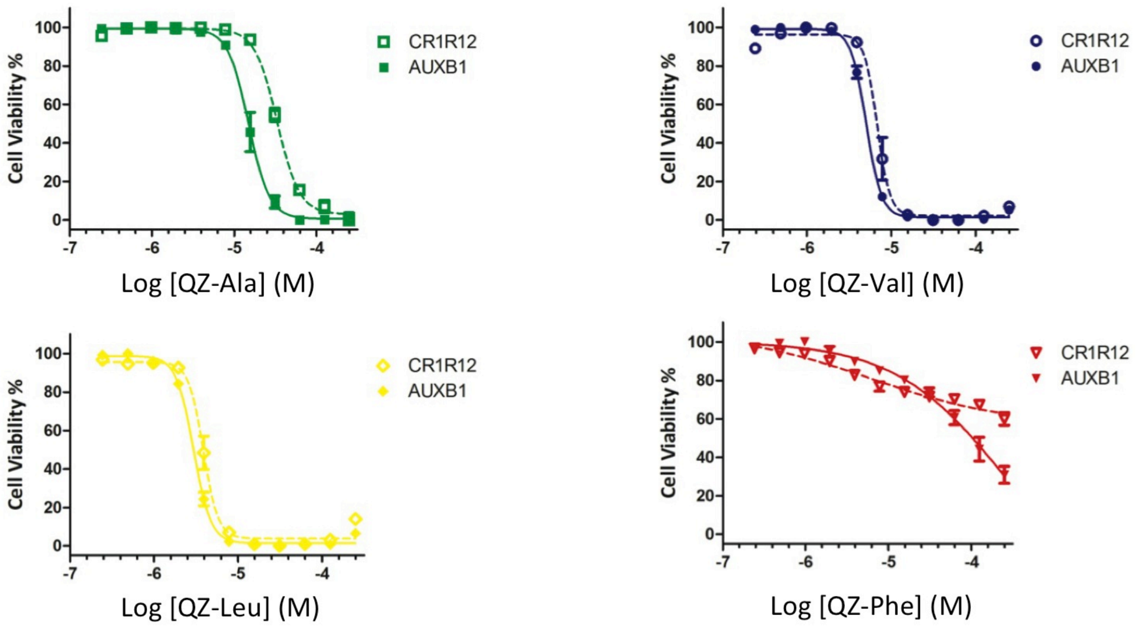

	toxicity (IC$_{50}$ μM) CR1R12 / AUXB1
QZ-Ala	32.1 ±0.9 / 14.8±0.5
QZ-Val	7.1±0.3 / 5.3±0.1
QZ-Leu	3.9±0.1/ 3.1±0.1
QZ-Phe	>100 μM/ ~60 μM

Supplementary Figure S3

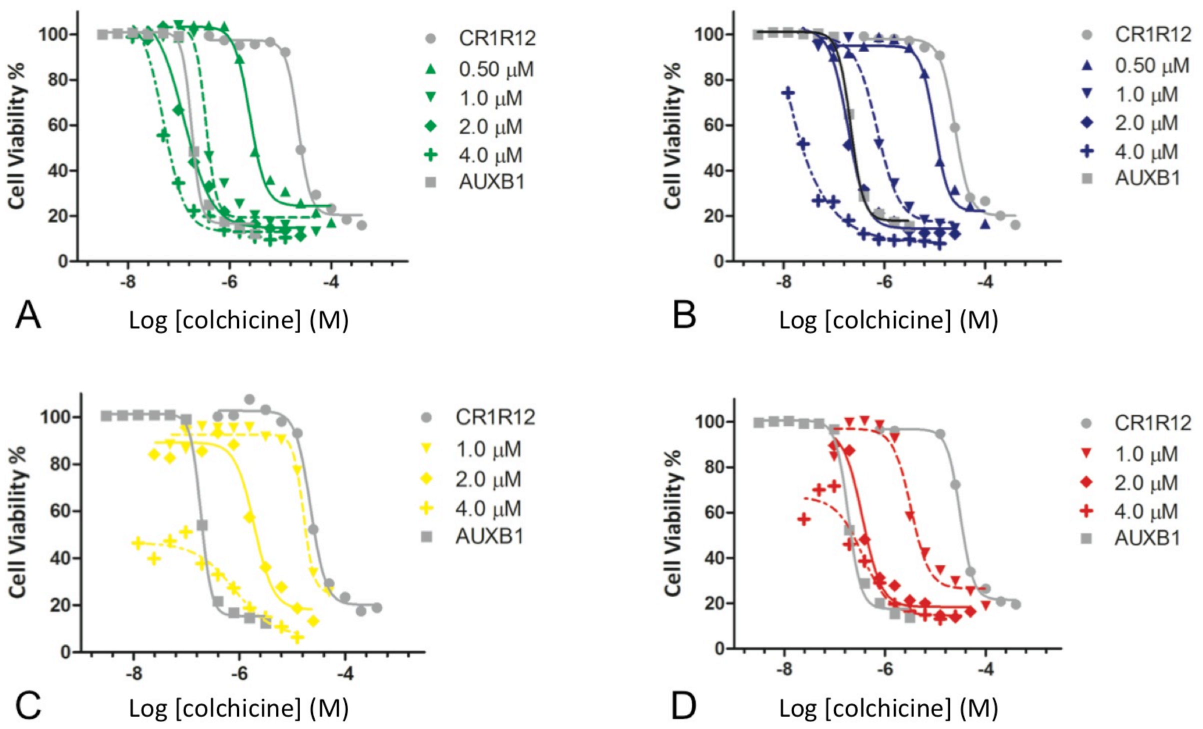

Supplementary Figure S4
